# Rescue and Emergency Management of a Man-Made Disaster: Lesson Learnt from a Collapse Factory Building, Bangladesh

**DOI:** 10.1155/2015/136434

**Published:** 2015-04-14

**Authors:** Animesh Biswas, Aminur Rahman, Saidur Rahman Mashreky, Tasnuva Humaira, Koustuv Dalal

**Affiliations:** ^1^Centre for Injury Prevention and Research, Bangladesh (CIPRB), Dhaka, Bangladesh; ^2^Centre for Injury Prevention and Safety Promotion, Department of Public Health Sciences, School of Health and Medical Sciences, Örebro University, 701 82 Örebro, Sweden

## Abstract

A tragic disaster occurred on April 24, 2013, in Bangladesh, when a nine storied building in a suburban area collapsed and killed 1115 people and injured many more. The study describes the process of rescue operation and emergency management services provided in the event. Data were collected using qualitative methods including in-depth interviews and a focus group discussion with the involved medical students, doctors, volunteers, and local people. Immediately after the disaster, rescue teams came to the place from Bangladesh Armed Forces, Bangladesh Navy, Bangladesh Air Force, and Dhaka Metropolitan and local Police and doctors, medical students, and nurses from nearby medical college hospitals and private hospitals and students from colleges and universities including local civil people. Doctors and medical students provided 24-hour services at the disaster place and in hospitals. Minor injured patients were treated at health camps and major injured patients were immediately carried to nearby hospital. Despite the limitations of a low resource setting, Bangladesh faced a tremendous challenge to manage the man-made disaster and experienced enormous support from different sectors of society to manage the disaster carefully and saved thousands of lives. This effort could help to develop a standard emergency management system applicable to Bangladesh and other counties with similar settings.

## 1. Introduction

Building collapses are a major cause of mortality and morbidity around the world. In the last decade, a number of buildings have collapsed causing a significant number of deaths and illness. In recent years, high and middle income countries like USA, South Korea, Turkey, Austria, and China have experienced disasters due to collapsed buildings [1–6]. In New work City, six people were killed and more than 20 were injured in two residential buildings which collapsed due to gas explosion [1]. Another six people were injured in a building site collapse at the new Turkish railway in May 2014 [2]. North Korea faced a 23 storied residential building collapse and in South Korea there was also a similar type of building collapse, which killed 10 people including nine university students with many more [2, 3]. India has experienced a number of recent deadly building collapse disasters. During 2014, the country had two major building disasters, which occurred in Mumbai in April 2014 killing 45 people including 15 children and another 70 people were injured in the incident [7]. Similarly, Bangladesh has also experienced such man-made disasters during the last decade, which have caused a large number deaths and injuries.

On April 24, 2013, a nine-story building named Rana Plaza situated at Savar subdistrict in Dhaka suddenly collapsed around 9 o'clock in the morning (Figure 3). The building contained four garment industries, a bank, apartments, and several other shops. This man-made disaster caused a massive loss of human and material, economic, and environmental loss. Although Bangladesh has experienced similar disasters previously, no other such disasters were so serious considering the death toll. It has been considered to be the deadliest garment-factory collapse in the history of Bangladesh, as well as the deadliest accidental structural failure in modern human history [8]. Local volunteers with the help of the trained forces were deployed to rescue the victims, whether dead or alive (Figure 4). Unsafe human act took this greatest toll, but the same mankind came forward to save hundreds of lives from the terrible event. This paper explores how the rescue process took place and the initial emergency medical management of the victims at the event by trained forces and untrained community rescuers and its lesson learnt.

## 2. Materials and Methods

### 2.1. Location of the Disaster

Savar, a subdistrict, is located about 25 kilometers northwest of the capital city Dhaka, Bangladesh. It has a population of over 13 million. The subdistrict has an industrial importance, particularly due to the presence of garments industries. Garment industries are widely located and many thousands of people in the workforce earn their livelihood by their hard work in these industries. The subdistrict is well connected with Dhaka by road. There is one military cantonment, one public university, and two private medical college hospitals with good health facilities [9].

### 2.2. Gathering Information

We deployed the following techniques to gather relevant information related to rescue and emergency management:

- reviewing printed and electronic media reports including newspaper and television coverage;
- in-depth interviews with the rescuers and personnel involved in emergency medical management;
- focus group discussions (FGD) with undergraduate medical students who assisted in emergency management.

#### 2.2.1. Reviewing Print and Electronic Media Reports

After the event, we reviewed five daily popular local language (Bengali) and three English newspapers from April 24, 2013, to the end of rescue operation, May 13, 2013. We also followed the news of electronic media (television) to get information and updates of the events and noted the relevant information.

#### 2.2.2. In-Depth Interviews

To gain deeper understanding we performed in-depth interviews (IDIs) with different personnel. We have performed in-depth interviews (IDIs) with different personnel who were directly involved in rescue and emergency medical management immediately after the event. The interviewees were with two medical doctors, two scout volunteers, and two local untrained volunteers. The in-depth interviews were conducted over the telephone and each interview took around 20–30 minutes. The interviews were conducted by an anthropologist within the research team.

#### 2.2.3. Focus Group Discussions

Moreover, one focus group discussion (FGD) was conducted with seven undergraduate medical students studying in a private medical college named Gonoshasthaya Samaj Vittik Medical College situated in the subdistrict. These students assisted in emergency management of the victims in the health camp at the scene (Figure 5). The FGD was organized at the medical college on May 20, 2013. The discussion continued for about 90 minutes. The interviewing team consisted of a public-health specialist and a facilitator.

### 2.3. Checklist for IDIs and FGD

A semistructured checklist was developed and used to conduct IDIs and the FGD. In the checklist seven thematic areas of discussion were identified, which included understanding of the event, initiation of the emergency management, engagement of the media, referral of the injured victims, hospital administrators response to the disaster, challenges encountered during emergency management, and finally the effectiveness and achievement of the emergency management. To explore each of the discussion areas, several prompts were used (Table 1).

### 2.4. Data Collection and Analysis

In case of IDIs the telephone interviews were audio-recorded with prior permission from the interviewees and hand notes were taken for some of the thematic areas during the telephonic conversation. The facilitator of the FGD session used prompts to explore the desired information, and similar to the IDIs this session was also audio-taped with the respondents' permission.

After the interviews and discussion, a two-member team under the guidance of the researchers prepared Bengali transcripts of the audio records. Then the transcripts were translated into English.

The analysis was done by examining the transcripts and note-taker's notes in detail to identify the range of ways in which the participants responded to various prompts. Thematic analysis was performed for FGD and IDIs whereas content analysis was made for analyzing electronic and printing media news.

## 3. Results

We have designed the Results section according to the areas that we identified for the IDIs, and FGD and content analysis from the media has also been incorporated.

### 3.1. Extent of Damage

Media reports revealed that the Rana Plaza, a nine storied building that housed four garment factories, a bank branch, and several general shops, collapsed at 9 a.m. on April 24, 2013. The garments factories were opened on their regular working hour at 8 o'clock in the morning. All the workers of the garment factories and general shops started their regular work in the building. All the machines and generators were switched on. Suddenly, pillars of the building collapsed and the roofs from the top to the bottom floors fell one upon another. Concrete pillars of every shape dangled precariously, ready to snap, and crashed down. Various media reported that the day before the event it was noticed that there were a number of cracks in pillars; however, the building's safety was assured by its owner.

Regarding building collapse one of the volunteers who participated in IDI narrated:

*The building (Rana Plaza) collapsed all on a sudden after a big bang around 9.00 a.m…hundreds of people died instantly; thousands injured, many of them trapped inside the collapse building alive.*

Another volunteer narrated his experiences in IDI:

*I have seen most of the floors of the building collapsed and…gaps between each of the roof of different floors looked no more than two feet. The nine storied building became three storied in height…No one could estimate actually how many people were trapped inside…we were so shocked to see this.*

### 3.2. Initiation of Rescue and Emergency Medical Management

**Figure 1:**
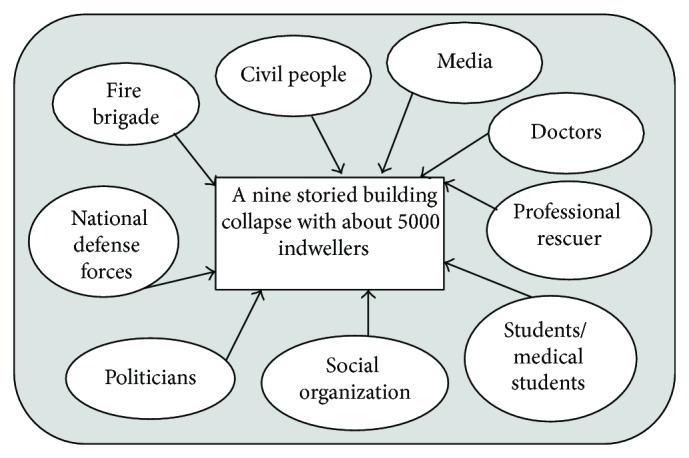
Responses just after the Savar disaster occurred.

As reported by different media, the Fire Service and Civil Defense (public fire-fighting authority) arrived at the scene within 30 minutes. Local people also came forward hearing the call for help by the victims who were trapped in the collapsed building. Fire Service and Civil Defense personnel with the help of local people rescued the first person within 30 minutes of the event. Immediately after the collapse other agencies including Police, Rapid Action Battalion (a special force of a combination of military and police personnel), Bangladesh Armed Forces, and Ansar (an aid to regular police force) came to the scene to help. At the beginning of the rescue process, about 200 Fire Service and Civil Defense personnel and around 200 local people participated in the rescue process. Several private ambulance services sent their ambulances voluntarily for transportation of victims to the nearby hospitals. Doctors and undergraduate medical students of different medical colleges of the locality arrived at the scene and set up medical camps to provide emergency services to the victims. Every few minutes for the first few hours of the disaster, seriously injured patients were rescued and emergency care was given by the medical teams at the camps. They were then carried to the nearby hospitals for further management. Red Crescent and volunteers from different organizations brought the patients from the damaged building to the health camps. Some victims were directly sent to the health facilities after the local people rescued them. Some volunteers and law enforcing agencies cleared the highway for immediate transfer of the patients (Figure 1).

A medical student that worked in the health camp described the following:

*When we came about an hour later, we saw that in every few minutes a trapped person is pulled out and rushed to the hospital in an ambulance or in microbus.*

One of the medical students who worked in the health camp narrated:

*We set-up our health camp within an hour (after the disaster). At the beginning, we were not prepared much, but we tried to treat minor injuries, the majority of casualties were directly sent to a nearby private hospital.*

One local volunteer described the following:

*Just after the incident, we tried to rescue people from inside the collapsed building and if we saw any person with blood shedding or broken leg or hand, we put them on the patient trolley…and people waiting outside took the victims immediately to the hospital. Many of the victims were even taken to the hospital by motorcycles of the local people.*

Another volunteer reported the following during his IDI:

*Bangladesh Army and police were handling the transferring patients with the support of the local people. There was minimum delay in transferring patients just after rescuing from the building.*

### 3.3. Engagement of Media

Media played a crucial role after the disaster. National TV channels including Bangladesh Television (BTV) and around 12 private TV channels, government radio station Bangladesh Betar, and FM stations started broadcasting live news from the start of the tragic event. Private TV channels scrolled the breaking news and special news was telecasted at regular intervals. A number of TV channels live telecasted from the spot the whole day. Media helped a lot to inform the relatives of those who worked in that building and many of the relatives started to come to Dhaka from villages just after getting information.

During an interview a doctor said:

*We took support from them to let the blood donor come to donate blood. We had requirement of thousands bags of blood and some of those had rare negative groups. *

One volunteer said during IDI:

*I saw the disaster event in television channel; my home is about 100 kilometers from the disaster place. As soon I get the information, me and two of my friends decided to come to the place and help in the rescuing.*

The story of one person who came from a rural village mentioned that he had come from north Bengal, which is about 180 kilometers from the disaster site. He saw that many people were coming to help the victims and rescuers by providing medicines, food, water, and so forth. He collected bananas from his own banana tree and bought packets of bread and caught a bus to Savar, which took about seven hours.

Another doctor mentioned that he found that a number of negative blood bags had been collected in the blood donation camp. The need for rare blood groups was transmitted by the television channels.

Due to the continuous news updates, many people started to come to the place to help and join the rescue process. Like this, many people also provided support and aid by knowing the facts through live telecasts and radio news updates. Moreover, the printed media detailed the entire situation including regular updates on how many people were rescued, how many were still missing, and how many of them had already died. People also read that news from newspapers and came to help the injured people. A few printed newspapers started a fund for the victims and many people donated money to the newspapers' bank account.

### 3.4. Referral System of the Patients

**Figure 2:**
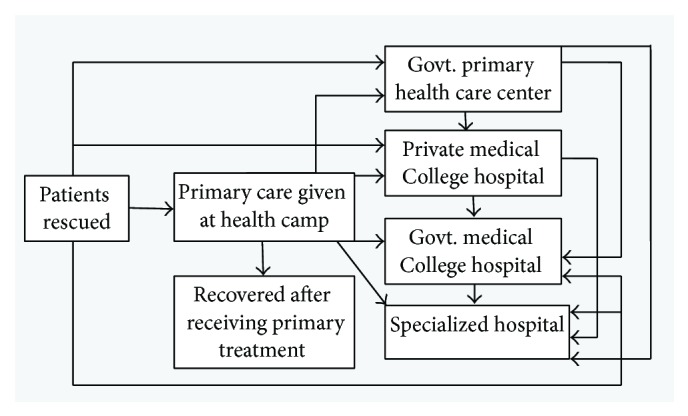
Referral system practiced during the Savar disaster.

**Figure 3:**
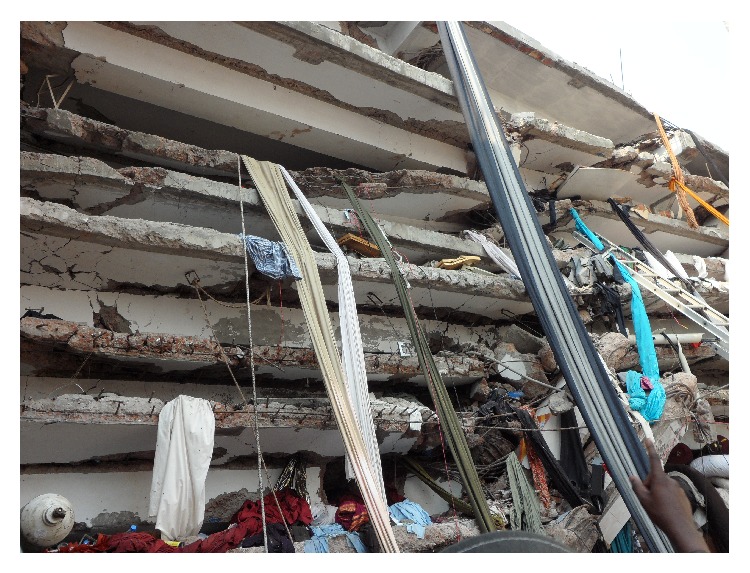
What happened after Rana Plaza collapsed.

**Figure 4:**
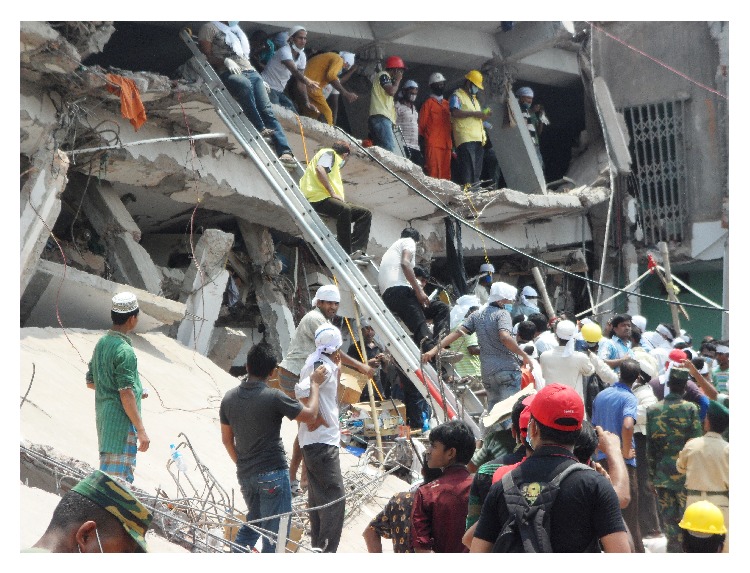
Rescue at Rana Plaza collapse.

**Figure 5:**
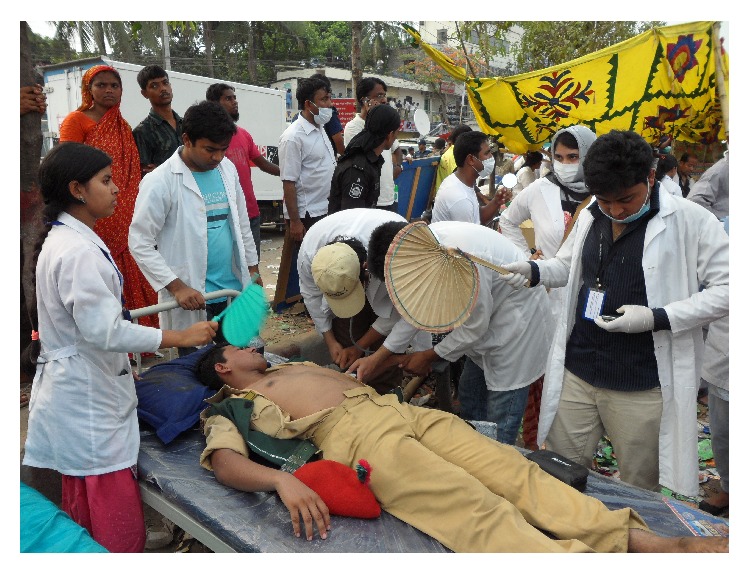
Treatment at make-shift camp after Rana Plaza collapsed.

Victims rescued from the disaster were primarily treated just outside of the collapsed building. There were two health camps engaged in providing 24-hour first aid treatment. Minor cut injuries were managed locally and released after initial treatment given at the center. Each of the health camps was equipped with first aid medicines and instruments. Doctors were responsible for immediately diagnosing the severity of the condition of the injured and referred to the appropriate health care facilities. However, some patients were directly transferred to referral centers without treatment from the health camps because of the degree of severity (Figure 2).

During in-depth interviews, one doctor who worked in the health camp mentioned the following:

*We provided initial treatment, many patients come to us with cut injuries, we cleaned the wound and put bandage. We also found huge number of patients with fractures; we provided first aid of fracture and referred them to the hospital immediately. *

He added

*We treated not only the people trapped in that building but also the rescuers who became sick during the rescuing. They had dehydration and became injured. *

During the FGD a medical student working at that health camp reported:

*We didn't have any referral form or structured documents, our seniors examined the patients and if it was required to transfer to higher center immediately, local people or fire brigade or armed force brought them by ambulance. *

The general people engaged in the recue process mentioned the following:

*We can guess which patients were required to send to hospital directly because many of patients had cut their limbs during rescuing and had severe bleeding, they were transferred immediately. Those with minimum injuries were treated in health camps. *

One doctor who worked in the private medical college hospital where most of the patients were transferred was describing his experiences:

*I never faced such conditions, patients were coming in every minute, lots of people were coming with patients. We don't have such emergency management system in the hospital but we are committed to tried at best level to manage such condition, our all operation theaters were in action 24 hours.*

A university student who volunteers on behalf of Bangladesh Scouts described the following:

*I found a 22 years old person was trapped behind a concrete beam, his leg was locked in between the beam, there was no way to remove the large beam, the person wanted to be rescued anyway, I used sharp blade to cut his left knee joint and rescued from the hole, immediately I pull him outside and ambulance took him away to government medical college hospital.*

### 3.5. Disaster Response of the Hospital Administrators

The disaster occurred away from the capital. There were two private medical college hospitals nearby and a number of private clinics and hospitals were also present. Savar Upazila has a government primary level health care center, Upazila health complex. Dhaka Medical College Hospital is about 30 kilometers from Savar and specialized hospitals like the orthopedic hospital is about 25 kilometers away from Savar. However, the Bangladesh Army has a specialized hospital in Savar near to where the event occurred. As soon as the disaster happened, different hospitals sent ambulances for transferring critical patients to the health centers. Private clinics also contributed by providing ambulances. One private medical college hospital set up an emergency care camp at the site within a few hours of the event, and the medical camp served until the last day of the rescue operation. Another private medical hospital's administration decided to provide services 24 hours a day and all the medical students of the hospital were engaged in the initial management of the casualty. Orthopedic specialized hospitals in Dhaka established a 24-hour emergency wing only for the patients from the Savar disaster. Private clinics in Savar managed primary stages of casualty care and then referred them to higher centers.

During FDG a medical student that worked at the health camp mentioned the following:

*Our medical college hospital administration allowed me to come to the health camp and we provide 24 hours support. Our health camp unit at the disaster center which existed till the last day of the rescue process.*

A doctor mentioned the following in IDI:

*I am doing internee in the private medical college in Savar and our director of the hospital was always with us and we were waiting for patients at emergency ward. Our medical students, nurses, word boys-everyone were engaged only in managing emergency situation. *

### 3.6. Challenges during Emergency Management

As a low income country, Bangladesh has very limited resources to manage a large scale emergency situation. The country has never experienced this type of disaster before. A number of challenges were faced during the rescue operation. Crowd management was crucial; there were thousands of people who came to see what was going on; many of them were relatives of the victims and they were waiting with apprehension to see if their relatives were alive and needed to be rescued or not. Moreover, another mass of people came to help the rescuers and rescued persons by providing food, medicine, and other necessities. Bangladesh Armed forces, Civil Aviation, Rapid Action Battalion, and Police played a tremendous role in managing the crowd. However, there was always a risk of mishap or an accident at any time. The majority of the public did not have any idea or training on how to rescue people from inside the building.

A volunteer said, “It was dark all around and I have seen many people crawling around in the light of their mobile phones. There was lot of dust from the collapsing debris, worsening smell of dead bodies were all around, inside the trap was hot because of full summer season and deficit of air ventilation.”

Rescue operations had proven to be thoroughly difficult. The pillars and ceilings had collapsed at so many angles and in such precarious ways that any wrong move could cause a fresh tragedy. The army had brought in huge cranes to pull the concrete blocks apart. But these could not be used earlier for the fear of further collapse. There were also limitations for not having necessary equipment to rescue people in such an emergency. Moreover, there was a lack of highly skilled rescuers to manage the critical time; those who are engaged in the rescue period were not properly trained on how to handle such conditions. However, members of the Armed Forces, Border Guard Bangladesh, and Fire Service and locals were carrying out the rescue operations; they pored over huge piles of rubble and twisted metal in the search for survivors at the building.

During a discussion with a medical doctor involved in providing emergency care, he mentioned the following:

*We don't have any training on emergency medical care, like this hospitals also don't have proper emergency management system. We are trying to do what we have learnt during our bachelor course but it's so important to learn in oder [sic] to handle such situations with enough skills.*

Moreover, in the FGD, one medical student mentioned the spirit of a civilian; he found a vegetable shop keeper who kept coming to the health camp for first aid treatment mentioning that he has a skill of how to climb in the trees and he used that skill in the rescue process. That person heard the screams of those who were trapped inside; some shout for help and others wanted some air; he had believed that he could rescue some people from inside and he contributed.

However, medical students working in the health camp said:

*Entire team had a challenge to liaison with different type of rescuers. Bangladesh army was leading the operation with the support of fire brigade but there was huge number of untrained civil people including students engaged in the rescue team.*

### 3.7. Strength and Achievement in Emergency Management

From in-depth interviews and FGDs it was revealed that, despite a lack of skilled manpower, proper training, necessary equipment, and a number of other challenges, the country expressed huge strength in managing such a devastating situation. Thousands of people from the local area and around it came to join the rescue teams. Battalions from Bangladesh Army, about 200 highly skilled fire fighters, about 275 trained volunteers, 100 young rovers scouts, 100 young red crescent volunteers, students from universities and colleges, medical students, doctors, nurses, volunteers of social organizations, volunteer clubs, and social workers put their efforts as a team to minimize the number of casualties and fatalities. Thousands of blood bags were collected from different parts of Dhaka city and near to Dhaka districts through blood donation camps. Hospitals provided free services for all injured patients; specialized doctors operated on critical cases immediately after admission into the hospitals. Furthermore, the media was telecasting news using live telecast which also helped civil people to know and act quickly. Sometimes they knew from the breaking news that there was certain shortage of a specific blood group at a hospital or that there was unavailability of ambulances to carry patients to referral centers. Bangladesh Armed Forces opened a monitoring cell to provide continuous updates about the situation. Every hour the data base in that cell was updated and people came to know how many persons were rescued, how many of them died, and how many were still missing.

When talking to medical students during the FGD, the following was mentioned:

*We never seen the liberation war of Bangladesh, it was about 42 years back, we were not even born at that time. But for me it's a war, the difference between the two is last time we fight for the entire nation, this time we are fighting to save thousands of lives.*

During the entire time, people had a commitment, spirit, braveness, and moral courage for the humanity. Many of the rescuers were injured during the rescue operation but they did not stop searching.

The achievement was so astonishing that one of the medical students stated:

*One of my batch mate studying in 5th year; was too brave, rescued one woman by cut her knee and recued in between two pillars on 5th floor.*

Good examples of humanity and dedication were remarkable. In the daily national Bengali newspaper, Prothom Alo wrote a number of articles about rescuing people alive, including May 1, 2013, stating that four fire fighters gambled with their lives to rescue others who were working for more than 15 years in Fire Brigade. One of them was burnt during a rescue but they altogether saved about 143 people's lives. Garments workers saved a number of people trapped inside believing they were their siblings and it was their responsibility to come forward to rescue them. Another garment worker saved about 24 persons; one of them was a 12-year-old girl and the rescuer cut the hand of that girl with a blade to help rescue her. A teacher of a religious school (madrasa) with the support of others saved about 150 people. He did not know any other rescuers and did not ask for their names even. Their one and only aim was to save people from the building.

An outstanding success achieved was reported by BBC online news on May 10, 2013, when a woman was rescued after 17 days of the operation when she was found in the remains of the second floor of the Rana Plaza. The worker who first discovered her told the BBC Bengali service that he was cutting iron rods and suddenly found a silvery stick just moving from a hole. He looked closer and heard someone calling for help. The volunteer immediately called over soldiers and firefighters to rescue the woman. When the woman was rescued it was like a miracle as she had been trapped for such a long time [10].

After 20 days of the rescue operation, the Daily Sun, an English newspaper in Bangladesh, on May 14, featured that Bangladesh retrieved 1,115 dead bodies from the debris and 2,438 rescued alive including 1,744 injured from the rubble of Rana Plaza. However, unfortunately, a total of 12 workers died after they were rescued with injuries. A total of 98 people were missing until the final phase of the rescue operation [11].

## 4. Discussion

Bangladesh has already experienced a number of building collapse tragedies. Even though the magnitudes of these types of disasters were not as great as the one of the Rana Plaza disaster, they also took a toll on humanity. During the review of daily newspaper articles on building collapses, we have found a number of incidents occurring in the last decade in Dhaka. Among those, on June 9, 2004, one three storied building collapsed in old Dhaka city where 19 people died and 11 were seriously injured. Another one was a garment factory; a nine-story building collapsed in Savar on April 11, 2005, killing 64 people working there. Likewise, a five-story building inside the capital city Dhaka suddenly collapsed on February 25, 2006, and death took its toll on 21 innocent people. Moreover, a residential building collapsed on July 1, 2010, in a slum area of Dhaka killing 25 people. Other types of sudden disasters include launch drowning's in Chandpur killing over 800 people in 2003, a fire in a garment factory killing 124 innocent workers on November 24, 2012, a collapse of a flyover during construction killing dozens of men, and other such disasters that keep happening in Bangladesh. An alarming fact is that accidents in the waterways have killed 3,522 people in the last decade.

Emergency care is the essential basis for all-hazards disaster responses [12]. Many countries manage natural disaster effectively. New Zealand experienced a 6.3 magnitude earthquake causing buildings collapses and rubble to fall on the road and injure thousands. Staff with equipment was mobilized to the open air ambulance bay to provide triage and initial care [13]. China managed an earthquake of 7.1 magnitudes by evacuating people by air to hospitals and relief work started within two hours [14].

However, emergency medical systems are a critical component of national health systems in low and middle income countries [15]. In countries like Bangladesh, management of a disaster is difficult because of various obstacles and challenges. However, during the Rana plaza disaster, an entire nation worked together to rescue and initiate emergency management. Efforts from the general people who did not have any training on emergency management took accountability. Like this, medical students of different levels provided first aid and prehospital care.

Armed Forces, volunteers, and local people transferred injured patients to hospital by assessing the severity of casualties but there was no proper referral system. Different types of vehicles were used to transfer patients to hospitals including ambulances, private microbus, motor bike, and nonmotorized vehicles, but this kind of support is not unique for other disasters that have happened in Bangladesh. In EMS, transporting a patient from the location to a hospital is a critical element of prehospital care, since a lack of transportation or suitable transportation is often the major barrier preventing patients from accessing emergency care [16]. In Rana plaza, where motor bikes were used to transfer patients and those bikes were fitted with a special horn to produce sound, this type of patient transfer is already in practice in many countries [17].

Like this, in nearby private medical hospitals where most of the patients were carried to, the hospital administration gave all of their efforts to manage the situation. All nurses of the hospitals, medical college students, intern doctors, and other doctors were mostly engaged in managing the disaster casualties. At a hospital level there was no structured emergency medical management system too. One study identified that some of the barriers to emergency care at the receiving health facilities were human factors, such as the availability of trained health workers; structural factors, such as availability of medications and equipment; and management factors, including mechanisms for the triage of patients [18], while in case of a mass casualties there is the need for adequate preparedness, including planning and training, a need to manage casualty flow through triage, effective team structure, and a need for improved communication and use of specific interventions [19–22]. During the Rana plaza disaster the country experienced the handling of such a major incident, planned instantly, and worked as a team with a goal to maximize the number of lives to be saved. Different skilled and nonskilled groups worked together; most of them came for the first time in their life to rescue people; they took the challenges and saved lives.

## 5. Conclusions

Despite limited resources the trained and untrained rescuers and the service providers of different sectors provided tremendous support for saving lives in the Rana Plaza tragedy; otherwise the toll could have been much more. This tragedy gives us a lesson that the nation needs to develop a structured comprehensive emergency management system involving all sectors, including lay people.

## Figures and Tables

**Table 1:** Checklist for IDIs and FGD.

Qualitative method used and group of the participants with age range	Area of discussion	Prompt used
(1) FGD ( aged 21–25 years), 7 persons Medical students (2) IDI (25 yrs–45 yrs ) Medical doctor, volunteer, general people	Extent of damage	(i) What happened? (ii) How did it happen? (iii) What was level of damage?
	Initiation of rescue and emergency management	(i) How were rescue and emergency management initiated? (ii) Who were involved?
	Engagement of media	(i) How did media act? (ii) What was the impact of media on people?
	Referral of the injured patients	(i) What was the process to referral system? (ii) How were the patients transferred? (iii) Who were involved in the process? (iv) What was the role of different people involved in the process?
	Disaster response of the hospital administrators	(i) What was the preparedness at the hospital? (ii) How were hospital administrators acting?
	Challenges during emergency management	(i) What were the challenges faced during rescue and emergency medical management?
	Strength and achievement of emergency management	(i) What were the strengths? (ii) What were the achievements noted? (iii) Is there any success stories?

## References

[1] (2014). Six dead in New York City buildings collapse. *BBC News*.

[2] http://www.bbc.com/news/world-asia-27459186.

[3] http://www.reuters.com/article/2014/02/18/us-korea-collapse-idUSBREA1G1HE20140218.

[4] Toksabay E. (2014). Six injured in building site collapse on new Turkish railway. *Reuters*.

[5] http://www.independent.co.uk/news/world/europe/several-injured-as-building-collapses-after-explosion-in-vienna-9291148.html.

[6] http://www.telegraph.co.uk/news/worldnews/asia/china/10743869/Building-collapses-in-China-rescuers-search-for-residents.html.

[7] http://www.bbc.co.uk/news/world-asia-22046466.

[8] http://www.bbc.com/news/world-asia-22394094.

[9] http://en.wikipedia.org/wiki/Savar.

[10] http://www.bbc.co.uk/news/world-asia-22477414.

[11] http://www.daily-sun.com.

[12] Marghella P. D. (2005). Surge capacity planning in health care organizations: hitting the mark on enhancing national preparedness. *Homeland Defense Journal*.

[13] Ardagh M. W., Richardson S. K., Robinson V. (2012). The initial health-system response to the earthquake in Christchurch, New Zealand, in February, 2011. *The Lancet*.

[14] Liu X., Liu Y., Zhang L. (2013). Mass aeromedical evacuation of patients in an emergency: experience following the 2010 yushu earthquake. *The Journal of Emergency Medicine*.

[15] Kobusingye O. C., Hyder A. A., Bishai D., Hicks E. R., Mock C., Joshipura M. (2005). Emergency medical systems in low- and middle-income countries: recommendations for action. *Bulletin of the World Health Organization*.

[16] Levine A. C., Presser D. Z., Rosborough S., Ghebreyesus T. A., Davis M. A. (2007). Understanding barriers to emergency care in low-income countries: view from the front line. *Prehospital and Disaster Medicine*.

[17] Soares-Oliveira M., Egipto P., Costa I., Cunha-Ribeiro L. M. (2007). Emergency motorcycle: has it a place in a medical emergency system?. *The American Journal of Emergency Medicine*.

[18] Donahue A. K., Tuohy R. V. (2006). Lessons we don't learn: a study of lessons on disasters, why we repeat them and how we can learn them. *Homeland Security Affairs*.

[19] Bradt D. A., Aitken P., FitzGerald G., Swift R., O'Reilly G., Bartley B. (2009). Emergency department surge capacity: recommendations of the Australasian surge strategy working group. *Academic Emergency Medicine*.

[20] Greenwood J. E., Pearce A. P. (2006). Burns assessment team as part of burn disaster response. *Prehospital and Disaster Medicine*.

[21] Palmer D. J., Stephens D., Fisher D. A., Spain B., Read D. J., Notaras L. (2003). The bali bombing: the royal darwin Hospital response. *Medical Journal of Australia*.

[22] Broeze C. L., Falder S., Rea S., Wood F. (2010). Burn disasters—an audit of the literature. *Prehospital and Disaster Medicine*.

